# Monoclinic polymorph of 2,5-dide­oxy-2,5-epithio-1,3:4,6-bis-*O*-[(*R*)-phenyl­methyl­ene]-l-iditol^1^

**DOI:** 10.1107/S1600536812034514

**Published:** 2012-08-11

**Authors:** Jerrell G. Gibson, Jung Young Cho, Frank R. Fronczek, Steven F. Watkins

**Affiliations:** a Department of Chemistry, Louisiana State University, Baton Rouge, LA 70803-1804, USA

## Abstract

The title compound C_20_H_20_O_4_S, is polymorphic. In the tetra­gonal form, the mol­ecule lies on a crystallographic twofold axis, while the monoclinic form has only approximate *C*_2_ mol­ecular symmetry. The greatest excursion from *C*_2_ symmetry is in the orientation of the two phenyl rings; at 100 K, one of the rings is rotated −37.2 (3)° and the other by 46.9 (3)° from their symmetric (tetra­gonal) positions. There are only minor differences in the three-ring nucleus; the best mol­ecular fit of the tetra­gonal and monoclinic forms, both at 100 K and excluding phenyl rings and H atoms, shows an r.m.s. deviation of 0.066 Å. Both forms have the same absolute configuration.

## Related literature

For details of the synthesis, see: Rao *et al.* (1988 ▶). For the structure of the tetra­gonal form at 295 K (CCDC refcode WAMRAD), see: Rao *et al.* (1993 ▶); and at 100 K (CCDC refcode 851380), see: Gibson *et al.* (2011 ▶). For a preliminary report of the structures of both polymorphs at room temperature, see: Fronczek *et al.* (2002 ▶). For a description of the Cambridge Structural Database, see: Allen (2002 ▶). For the determination of the absolute configuration from Bijvoet pairs, see: Hooft *et al.* (2008 ▶); Flack (1983 ▶). For details of the molecular mechanics software used, see: Cambridgesoft (2010 ▶); Winn & Goodman (2001 ▶).
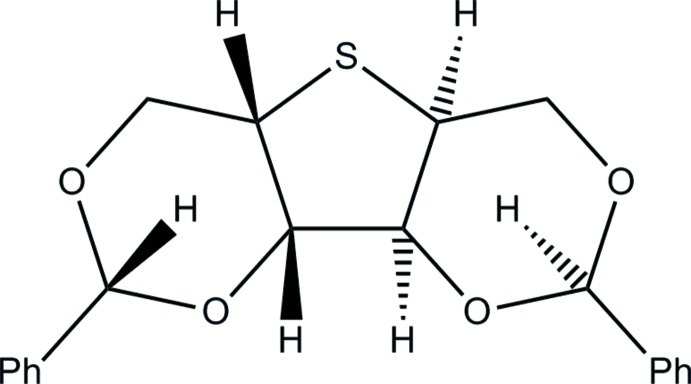


## Experimental

### 

#### Crystal data

C_20_H_20_O_4_S
*M* *_r_* = 356.42
Monoclinic, 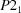

*a* = 6.158 (1) Å
*b* = 9.223 (2) Å
*c* = 15.407 (4) Å
β = 95.785 (8)°
*V* = 870.6 (3) Å^3^
*Z* = 2
Mo *K*α radiation
μ = 0.21 mm^−1^
*T* = 100 K
0.25 × 0.22 × 0.10 mm

#### Data collection

Nonius KappaCCD diffractometer
Absorption correction: multi-scan (*SCALEPACK*; Otwinowski & Minor, 1997 ▶) *T*_min_ = 0.950, *T*_max_ = 0.980
6225 measured reflections
6225 independent reflections
5648 reflections with *I* > 2σ(*I*)

#### Refinement

*R*[*F* ^2^ > 2σ(*F* ^2^)] = 0.037
*wR*(*F* ^2^) = 0.088
*S* = 1.05
6225 reflections
227 parameters
1 restraint
H-atom parameters constrained
Δρ_max_ = 0.29 e Å^−3^
Δρ_min_ = −0.23 e Å^−3^
Absolute structure: Flack (1983 ▶), 2730 Bijvoet pairs
Flack parameter: 0.02 (5)

### 

Data collection: *COLLECT* (Nonius, 2000 ▶); cell refinement: *DENZO* and *SCALEPACK* (Otwinowski & Minor, 1997 ▶); data reduction: *DENZO* and *SCALEPACK*; program(s) used to solve structure: *SHELXS86* (Sheldrick, 2008 ▶); program(s) used to refine structure: *SHELXL97* (Sheldrick, 2008 ▶); molecular graphics: *ORTEP-3 for Windows* (Farrugia, 1997 ▶); software used to prepare material for publication: *WinGX* (Farrugia, 1999 ▶) and *IDEAL* (Gould *et al.*, 1988 ▶).

## Supplementary Material

Crystal structure: contains datablock(s) global, I. DOI: 10.1107/S1600536812034514/mw2079sup1.cif

Structure factors: contains datablock(s) I. DOI: 10.1107/S1600536812034514/mw2079Isup2.hkl

Additional supplementary materials:  crystallographic information; 3D view; checkCIF report

## Figures and Tables

**Fig. 1.**
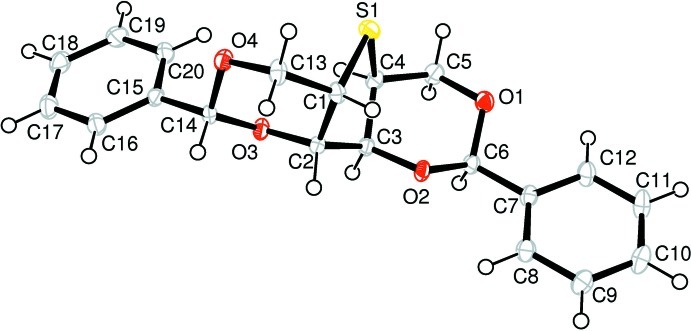
View of (I) (50% probability displacement ellipsoids).

## supplementary crystallographic information

### Comment

The tetragonal form of the title compound (I), C_20_H_20_O_4_S, was determined at 295 K (Rao *et al.*, 1993, hereinafter P4_295_). We re-examined the tetragonal form at 100 K in our laboratory (hereinafter P4_100_) and determined the absolute configuration. These data are deposited at the Cambridge Crystallographic Data Centre (Allen, 2002), CCDC 851380 (Gibson *et al.*, 2011). There is a 2.8% volume decrease upon lowering the temperature from 295 K to 100 K but the molecular symmetry is C_2_ at both temperatures and there is no significant change in molecular structure. The best molecular fit (Gould *et al.*, 1988) of all non-H atoms in P4_295_ and P4_100_ yields δ_r.m.s._ = 0.022 Å, while excluding the phenyl groups yields δ_r.m.s._ = 0.011 Å.

In the monoclinic form at 100 K (P2_100_) the molecular symmetry is C_1_. The molecular volume of P2_100_ (435.2 (3) Å^3^/molecule) is greater than P4_100_ (433.6 (1) Å^3^/molecule). The thirteen non-H atoms of the three central rings in P2_100_ and P4_100_ are arranged similarly with best molecular fit δ_r.m.s._ = 0.066 Å. The most pronounced structural difference between P2_100_ and P4_100_ is in the orientation of the phenyl rings. In P4_100_, two equivalent O—C—C—C torsions of -14.5 (3)° become +32.4 (2)° and -51.7 (2)° in P2_100_. The global minimum conformation of this molecule has been calculated using *MM2* (Winn & Goodman, 2001) and confirmed by us (Cambridgesoft, 2010). Like the tetragonal form, the minimum conformation has C_2_ symmetry, but the two equivalent O—C—C—C torsions representing the orientation of the phenyl rings are -56°.

Based on the Flack parameter *x* = 0.02 (5) (Flack, 1983) and Hooft parameters *y* = 0.00 (3) and P2(true) = 1.000 for 2730 Bijvoet pairs (Hooft *et al.*, 2008), the absolute configurations of the tetragonal and monoclinic forms are identical at 100 K, with stereochemical specification (2*R*,4aS,5aS,8*R*,9aS,9bS)-2,8-diphenylhexahydrothieno[3,2 - d:4,5 - d'] bis([1,3]dioxine).

### Experimental

The title compound was prepared by Dr. Ronald Voll according to the scheme reported by Rao *et al.* (1988). A suitable single-crystal was obtained by recrystallization from ethanol.

### Refinement

All H atoms were placed in calculated positions, guided by difference maps, with C—H bond distances 0.95 (aromatic C), 0.99 (CH_2_), 1.00 Å, (*R*_3_CH) and *U*_iso_=1.2*U*_eq_, thereafter refined as riding. The absolute configuration, based on 2730 Bijvoet pairs with Flack parameter *x* = 0.02 (5), Hooft parameter *y* = 0.00 (3) and Hooft P2(true) = 1.000, is consistent with that of the starting materials.

### Crystal data

**Table:**

C_20_H_20_O_4_S	*F*(000) = 376
*M**_r_* = 356.42	*D*_x_ = 1.36 Mg m^−^^3^
Monoclinic, *P*2_1_	Melting point: 481.5(5) K
Hall symbol: P 2yb	Mo *K*α radiation, λ = 0.71073 Å
*a* = 6.158 (1) Å	Cell parameters from 3254 reflections
*b* = 9.223 (2) Å	θ = 2.5–33.1°
*c* = 15.407 (4) Å	µ = 0.21 mm^−^^1^
β = 95.785 (8)°	*T* = 100 K
*V* = 870.6 (3) Å^3^	Lath, colorless
*Z* = 2	0.25 × 0.22 × 0.10 mm

### Data collection

**Table:**

Nonius KappaCCD diffractometer	6225 independent reflections
Radiation source: sealed tube	5648 reflections with *I* > 2σ(*I*)
Horizonally mounted graphite crystal monochromator	*R*_int_ = 0
Detector resolution: 9 pixels mm^-1^	θ_max_ = 33.1°, θ_min_ = 2.6°
ω and φ scans	*h* = −9→9
Absorption correction: multi-scan (*SCALEPACK*; Otwinowski & Minor, 1997)	*k* = −14→13
*T*_min_ = 0.950, *T*_max_ = 0.980	*l* = 0→23
6225 measured reflections	

### Refinement

**Table:**

Refinement on *F*^2^	Secondary atom site location: difference Fourier map
Least-squares matrix: full	Hydrogen site location: inferred from neighbouring sites
*R*[*F*^2^ > 2σ(*F*^2^)] = 0.037	H-atom parameters constrained
*wR*(*F*^2^) = 0.088	*w* = 1/[σ^2^(*F*_o_^2^) + (0.0366*P*)^2^ + 0.1562*P*] where *P* = (*F*_o_^2^ + 2*F*_c_^2^)/3
*S* = 1.05	(Δ/σ)_max_ = 0.001
6225 reflections	Δρ_max_ = 0.29 e Å^−^^3^
227 parameters	Δρ_min_ = −0.23 e Å^−^^3^
1 restraint	Absolute structure: Flack (1983), 2730 Bijvoet pairs
0 constraints	Flack parameter: 0.02 (5)
Primary atom site location: structure-invariant direct methods	

### Special details

**Table:**

Geometry. All e.s.d.'s (except the e.s.d. in the dihedral angle between two l.s. planes) are estimated using the full covariance matrix. The cell e.s.d.'s are taken into account individually in the estimation of e.s.d.'s in distances, angles and torsion angles; correlations between e.s.d.'s in cell parameters are only used when they are defined by crystal symmetry. An approximate (isotropic) treatment of cell e.s.d.'s is used for estimating e.s.d.'s involving l.s. planes.

### Fractional atomic coordinates and isotropic or equivalent isotropic displacement parameters (Å^2^)

**Table:**

	*x*	*y*	*z*	*U*_iso_*/*U*_eq_	
C1	0.7808 (2)	0.62050 (15)	0.17832 (8)	0.0169 (2)	
H1	0.7275	0.5554	0.1288	0.02*	
C2	0.6502 (2)	0.76146 (15)	0.17125 (8)	0.0163 (2)	
H2	0.4915	0.7418	0.1554	0.02*	
C3	0.7424 (2)	0.85423 (15)	0.10263 (8)	0.0161 (2)	
H3	0.681	0.9546	0.1024	0.019*	
C4	0.9911 (2)	0.85639 (15)	0.12534 (8)	0.0168 (2)	
H4	1.0276	0.9279	0.1734	0.02*	
C5	1.1053 (2)	0.90189 (16)	0.04642 (9)	0.0212 (3)	
H5A	1.086	1.0075	0.0369	0.025*	
H5B	1.2636	0.8822	0.058	0.025*	
C6	0.7929 (2)	0.84807 (15)	−0.04585 (8)	0.0167 (2)	
H6	0.7622	0.9546	−0.0476	0.02*	
C7	0.7107 (2)	0.78161 (14)	−0.13235 (8)	0.0172 (2)	
C8	0.4961 (2)	0.73435 (15)	−0.14903 (9)	0.0198 (2)	
H8	0.3999	0.7395	−0.1047	0.024*	
C9	0.4225 (2)	0.67920 (18)	−0.23124 (8)	0.0229 (2)	
H9	0.2771	0.6445	−0.2422	0.027*	
C10	0.5608 (2)	0.67481 (19)	−0.29699 (8)	0.0259 (3)	
H10	0.509	0.6391	−0.3531	0.031*	
C11	0.7744 (3)	0.72253 (17)	−0.28063 (9)	0.0279 (3)	
H11	0.8695	0.7192	−0.3255	0.033*	
C12	0.8496 (2)	0.77535 (16)	−0.19836 (9)	0.0232 (3)	
H12	0.9964	0.8073	−0.1872	0.028*	
C13	0.7489 (2)	0.54497 (15)	0.26421 (8)	0.0197 (2)	
H13A	0.5992	0.5049	0.2613	0.024*	
H13B	0.853	0.4633	0.2733	0.024*	
C14	0.6415 (2)	0.76351 (14)	0.32492 (8)	0.0161 (2)	
H14	0.4857	0.7313	0.3201	0.019*	
C15	0.6900 (2)	0.85965 (15)	0.40299 (8)	0.0168 (2)	
C16	0.5382 (2)	0.87628 (16)	0.46339 (9)	0.0204 (3)	
H16	0.4012	0.8282	0.4547	0.025*	
C17	0.5876 (3)	0.96367 (17)	0.53664 (9)	0.0252 (3)	
H17	0.4838	0.9756	0.5777	0.03*	
C18	0.7877 (3)	1.03315 (17)	0.54964 (9)	0.0255 (3)	
H18	0.8216	1.0916	0.5999	0.031*	
C19	0.9390 (2)	1.01740 (18)	0.48917 (9)	0.0263 (3)	
H19	1.0756	1.0658	0.4979	0.032*	
C20	0.8902 (2)	0.93077 (17)	0.41595 (9)	0.0225 (3)	
H20	0.9937	0.92	0.3746	0.027*	
O1	1.02017 (15)	0.82585 (11)	−0.03065 (6)	0.01980 (19)	
O2	0.68339 (15)	0.78354 (10)	0.02114 (5)	0.01686 (18)	
O3	0.68371 (15)	0.84402 (11)	0.24980 (6)	0.01752 (18)	
O4	0.78222 (15)	0.64292 (10)	0.33617 (6)	0.01824 (19)	
S1	1.06220 (5)	0.67372 (4)	0.16652 (2)	0.01900 (7)	

### Atomic displacement parameters (Å^2^)

**Table:**

	*U*^11^	*U*^22^	*U*^33^	*U*^12^	*U*^13^	*U*^23^
C1	0.0184 (6)	0.0173 (6)	0.0159 (5)	−0.0023 (4)	0.0049 (4)	−0.0009 (4)
C2	0.0156 (6)	0.0206 (6)	0.0127 (5)	0.0006 (4)	0.0016 (4)	−0.0001 (4)
C3	0.0186 (5)	0.0169 (6)	0.0130 (5)	0.0019 (5)	0.0022 (4)	0.0002 (4)
C4	0.0191 (5)	0.0158 (6)	0.0153 (5)	−0.0015 (5)	0.0009 (4)	0.0016 (4)
C5	0.0204 (6)	0.0236 (7)	0.0196 (6)	−0.0057 (5)	0.0024 (5)	0.0012 (5)
C6	0.0190 (5)	0.0181 (6)	0.0135 (5)	−0.0011 (5)	0.0035 (4)	0.0022 (4)
C7	0.0232 (6)	0.0146 (6)	0.0139 (5)	−0.0006 (5)	0.0029 (4)	0.0016 (4)
C8	0.0241 (6)	0.0181 (6)	0.0176 (5)	−0.0016 (5)	0.0036 (5)	0.0002 (5)
C9	0.0290 (6)	0.0186 (6)	0.0203 (5)	−0.0035 (6)	−0.0009 (5)	−0.0014 (5)
C10	0.0401 (7)	0.0211 (6)	0.0165 (5)	−0.0059 (7)	0.0020 (5)	−0.0034 (6)
C11	0.0402 (8)	0.0282 (8)	0.0168 (6)	−0.0065 (6)	0.0107 (5)	−0.0039 (5)
C12	0.0278 (7)	0.0255 (7)	0.0175 (6)	−0.0051 (5)	0.0073 (5)	−0.0010 (5)
C13	0.0287 (7)	0.0148 (6)	0.0164 (5)	−0.0010 (5)	0.0070 (5)	−0.0002 (4)
C14	0.0169 (5)	0.0195 (6)	0.0123 (5)	0.0018 (4)	0.0037 (4)	−0.0004 (4)
C15	0.0203 (6)	0.0174 (6)	0.0127 (5)	0.0036 (5)	0.0009 (4)	0.0010 (4)
C16	0.0218 (6)	0.0214 (7)	0.0187 (6)	0.0012 (5)	0.0048 (5)	−0.0010 (5)
C17	0.0307 (7)	0.0272 (8)	0.0184 (6)	0.0033 (6)	0.0067 (5)	−0.0028 (5)
C18	0.0309 (7)	0.0258 (7)	0.0190 (6)	0.0045 (6)	−0.0013 (5)	−0.0060 (5)
C19	0.0222 (7)	0.0315 (8)	0.0244 (7)	−0.0006 (6)	−0.0018 (5)	−0.0061 (6)
C20	0.0200 (6)	0.0290 (7)	0.0189 (6)	0.0003 (5)	0.0032 (5)	−0.0045 (5)
O1	0.0184 (4)	0.0243 (5)	0.0173 (4)	−0.0027 (4)	0.0045 (3)	−0.0002 (3)
O2	0.0193 (4)	0.0209 (5)	0.0107 (4)	−0.0027 (3)	0.0031 (3)	0.0004 (3)
O3	0.0244 (5)	0.0167 (4)	0.0119 (4)	0.0027 (4)	0.0038 (3)	0.0003 (3)
O4	0.0243 (4)	0.0164 (5)	0.0139 (4)	0.0029 (3)	0.0017 (3)	−0.0001 (3)
S1	0.01709 (13)	0.01938 (15)	0.02103 (13)	0.00212 (12)	0.00436 (10)	0.00274 (12)

### Geometric parameters (Å, º)

**Table:**

C1—C13	1.5256 (18)	C9—H9	0.95
C1—C2	1.5266 (19)	C10—C11	1.386 (2)
C1—S1	1.8279 (13)	C10—H10	0.95
C1—H1	1	C11—C12	1.3928 (19)
C2—O3	1.4271 (15)	C11—H11	0.95
C2—C3	1.5144 (18)	C12—H12	0.95
C2—H2	1	C13—O4	1.4287 (16)
C3—O2	1.4288 (15)	C13—H13A	0.99
C3—C4	1.5360 (18)	C13—H13B	0.99
C3—H3	1	C14—O4	1.4100 (15)
C4—C5	1.5236 (19)	C14—O3	1.4211 (15)
C4—S1	1.8376 (14)	C14—C15	1.5000 (17)
C4—H4	1	C14—H14	1
C5—O1	1.4322 (16)	C15—C16	1.3925 (18)
C5—H5A	0.99	C15—C20	1.3928 (19)
C5—H5B	0.99	C16—C17	1.3952 (19)
C6—O1	1.4103 (16)	C16—H16	0.95
C6—O2	1.4195 (15)	C17—C18	1.386 (2)
C6—C7	1.5073 (18)	C17—H17	0.95
C6—H6	1	C18—C19	1.390 (2)
C7—C8	1.3907 (19)	C18—H18	0.95
C7—C12	1.3945 (19)	C19—C20	1.390 (2)
C8—C9	1.3974 (18)	C19—H19	0.95
C8—H8	0.95	C20—H20	0.95
C9—C10	1.3879 (19)		
			
C13—C1—C2	109.75 (11)	C11—C10—C9	119.92 (12)
C13—C1—S1	114.52 (9)	C11—C10—H10	120
C2—C1—S1	105.18 (9)	C9—C10—H10	120
C13—C1—H1	109.1	C10—C11—C12	119.93 (13)
C2—C1—H1	109.1	C10—C11—H11	120
S1—C1—H1	109.1	C12—C11—H11	120
O3—C2—C3	104.97 (10)	C11—C12—C7	120.40 (14)
O3—C2—C1	111.34 (10)	C11—C12—H12	119.8
C3—C2—C1	107.41 (11)	C7—C12—H12	119.8
O3—C2—H2	111	O4—C13—C1	111.31 (11)
C3—C2—H2	111	O4—C13—H13A	109.4
C1—C2—H2	111	C1—C13—H13A	109.4
O2—C3—C2	105.99 (10)	O4—C13—H13B	109.4
O2—C3—C4	111.45 (10)	C1—C13—H13B	109.4
C2—C3—C4	106.47 (10)	H13A—C13—H13B	108
O2—C3—H3	110.9	O4—C14—O3	110.58 (10)
C2—C3—H3	110.9	O4—C14—C15	107.30 (10)
C4—C3—H3	110.9	O3—C14—C15	107.83 (11)
C5—C4—C3	110.57 (10)	O4—C14—H14	110.4
C5—C4—S1	114.52 (10)	O3—C14—H14	110.4
C3—C4—S1	105.32 (9)	C15—C14—H14	110.4
C5—C4—H4	108.8	C16—C15—C20	119.77 (12)
C3—C4—H4	108.8	C16—C15—C14	120.53 (12)
S1—C4—H4	108.8	C20—C15—C14	119.69 (12)
O1—C5—C4	111.54 (11)	C15—C16—C17	119.85 (14)
O1—C5—H5A	109.3	C15—C16—H16	120.1
C4—C5—H5A	109.3	C17—C16—H16	120.1
O1—C5—H5B	109.3	C18—C17—C16	120.16 (14)
C4—C5—H5B	109.3	C18—C17—H17	119.9
H5A—C5—H5B	108	C16—C17—H17	119.9
O1—C6—O2	110.77 (10)	C17—C18—C19	120.07 (13)
O1—C6—C7	109.08 (11)	C17—C18—H18	120
O2—C6—C7	109.30 (11)	C19—C18—H18	120
O1—C6—H6	109.2	C18—C19—C20	119.97 (14)
O2—C6—H6	109.2	C18—C19—H19	120
C7—C6—H6	109.2	C20—C19—H19	120
C8—C7—C12	119.57 (12)	C19—C20—C15	120.17 (13)
C8—C7—C6	121.27 (12)	C19—C20—H20	119.9
C12—C7—C6	119.06 (12)	C15—C20—H20	119.9
C7—C8—C9	119.79 (12)	C6—O1—C5	109.91 (10)
C7—C8—H8	120.1	C6—O2—C3	110.49 (10)
C9—C8—H8	120.1	C14—O3—C2	112.91 (10)
C10—C9—C8	120.36 (13)	C14—O4—C13	111.41 (9)
C10—C9—H9	119.8	C1—S1—C4	94.89 (6)
C8—C9—H9	119.8		
			
C13—C1—C2—O3	47.28 (14)	O3—C14—C15—C16	129.50 (12)
S1—C1—C2—O3	−76.39 (11)	O4—C14—C15—C20	67.48 (15)
C13—C1—C2—C3	161.68 (10)	O3—C14—C15—C20	−51.66 (15)
S1—C1—C2—C3	38.02 (11)	C20—C15—C16—C17	−0.2 (2)
O3—C2—C3—O2	−172.01 (9)	C14—C15—C16—C17	178.65 (12)
C1—C2—C3—O2	69.39 (12)	C15—C16—C17—C18	−0.4 (2)
O3—C2—C3—C4	69.21 (12)	C16—C17—C18—C19	0.8 (2)
C1—C2—C3—C4	−49.39 (13)	C17—C18—C19—C20	−0.6 (2)
O2—C3—C4—C5	45.85 (15)	C18—C19—C20—C15	0.0 (2)
C2—C3—C4—C5	160.99 (11)	C16—C15—C20—C19	0.4 (2)
O2—C3—C4—S1	−78.37 (11)	C14—C15—C20—C19	−178.47 (13)
C2—C3—C4—S1	36.77 (11)	O2—C6—O1—C5	−66.02 (13)
C3—C4—C5—O1	−46.70 (16)	C7—C6—O1—C5	173.64 (10)
S1—C4—C5—O1	72.07 (13)	C4—C5—O1—C6	56.52 (14)
O1—C6—C7—C8	153.67 (12)	O1—C6—O2—C3	65.43 (13)
O2—C6—C7—C8	32.42 (17)	C7—C6—O2—C3	−174.35 (10)
O1—C6—C7—C12	−30.07 (16)	C2—C3—O2—C6	−170.15 (10)
O2—C6—C7—C12	−151.31 (12)	C4—C3—O2—C6	−54.71 (13)
C12—C7—C8—C9	0.9 (2)	O4—C14—O3—C2	60.93 (13)
C6—C7—C8—C9	177.15 (13)	C15—C14—O3—C2	177.96 (10)
C7—C8—C9—C10	−1.7 (2)	C3—C2—O3—C14	−169.74 (10)
C8—C9—C10—C11	1.4 (3)	C1—C2—O3—C14	−53.83 (13)
C9—C10—C11—C12	−0.3 (2)	O3—C14—O4—C13	−62.23 (13)
C10—C11—C12—C7	−0.5 (2)	C15—C14—O4—C13	−179.58 (10)
C8—C7—C12—C11	0.2 (2)	C1—C13—O4—C14	57.33 (14)
C6—C7—C12—C11	−176.16 (13)	C13—C1—S1—C4	−134.53 (10)
C2—C1—C13—O4	−49.24 (15)	C2—C1—S1—C4	−13.96 (9)
S1—C1—C13—O4	68.77 (13)	C5—C4—S1—C1	−134.60 (10)
O4—C14—C15—C16	−111.36 (13)	C3—C4—S1—C1	−12.90 (9)
